# *Ardisia japonica* (Thunb.) Blume and *Lespedeza cuneata* G. Don may treat chronic obstructive pulmonary disease by targeting HK2 and PTAFR

**DOI:** 10.3389/fmed.2025.1527632

**Published:** 2025-05-06

**Authors:** Xian Luo, Zheng Hang Ge, Shan Luo, Bo Li, Xun Zhou, Yi Yang, Yong Jiang, Tao Tan, Ke Lin Wang

**Affiliations:** ^1^The Second Affiliated Hospital of Guizhou University of Traditional Chinese Medicine, Guiyang, Guizhou, China; ^2^Guizhou Provincial Staff Hospital, Guiyang, Guizhou, China

**Keywords:** chronic obstructive pulmonary disease, active ingredients, network pharmacology, HK2, PTAFR

## Abstract

**Introduction:**

Previous studies have demonstrated the significant efficacy of *Ardisia japonica* (Thunb.) Blume (Zijinniu) and *Lespedeza cuneata* G. Don (Tiesaozhou) in alleviating cough and reducing phlegm. This study employed network pharmacology and bioinformatics approaches to identify key genes associated with Zijinniu and Tiesaozhou in chronic obstructive pulmonary disease (COPD), offering insights into potential therapeutic strategies.

**Methods:**

Data on COPD, along with the active ingredients and target genes of Zijinniu and Tiesaozhou, were utilized. By integrating the results of differential expression analysis and the target genes of these two plants, candidate genes were identified. Key genes were then confirmed through gene expression analysis in the GSE124180 and GSE42057 datasets. A nomogram was constructed based on these genes to assess COPD risk, followed by validation. Additionally, functional analysis, immune factor profiling, molecular docking, and reverse transcription-polymerase chain reaction (RT-qPCR) were performed.

**Results:**

HK2 and PTAFR emerged as critical genes for COPD treatment, exhibiting significantly elevated expression in COPD samples. RT-qPCR confirmed the significantly higher expression of HK2 (*P* = 0.0425) in COPD samples. These findings highlight the potential of HK2 and PTAFR as therapeutic targets for COPD. Functional analysis further indicated that HK2 and PTAFR were co-enriched in pathways such as the “chemokine signaling pathway” and “FC gamma R-mediated phagocytosis,” suggesting their involvement in immune responses. Immune factor analysis revealed strong correlations between these genes and various chemotactic factors (e.g., CCL23, CCL5), immunosuppressants (e.g., IDO1, CSF1R), immunostimulants (e.g., ICOS, CD28), chemokine receptors (e.g., CXCR1, CXCR2), and major histocompatibility complex (MHC) molecules (e.g., HLA-B). Molecular docking revealed favorable binding energies between HK2 and quercetin (−8.2 kcal/mol), and between PTAFR and daucosterol (−8.4 kcal/mol), suggesting their potential as effective compounds targeting key genes for COPD therapy.

**Conclusion:**

HK2 and PTAFR were identified as crucial genes in COPD, providing a solid theoretical foundation for future treatment strategies.

## 1 Introduction

Chronic obstructive pulmonary disease (COPD) is a prevalent and heterogeneous chronic respiratory disorder characterized by various pulmonary and extrapulmonary clinical features, along with comorbid chronic conditions, making it the third leading cause of death globally (1). Patients typically present with distal bronchiectasis, airway wall degeneration, and progressive airflow limitation due to abnormal pulmonary inflammatory responses. As inflammation intensifies, pathological changes such as ciliary dysfunction and increased mucus production may arise, leading to clinical symptoms like cough and sputum production (2). COPD primarily affects the elderly, with its incidence steadily increasing due to worsening air pollution and the aging population. As a disease marked by high morbidity, disability, prolonged duration, and numerous complications, COPD significantly impacts patient quality of life and imposes a considerable economic burden on both families and society (3).

Recent studies indicate that acute exacerbations of COPD are linked to heightened airway and systemic inflammation, as well as physiological alterations (4). However, clinical management of COPD remains challenging due to the absence of specific treatments. Some studies show challenges to Western medicine’s effectiveness in treating common COPD (5, 6). COPD patients not only face the challenge of long-term disease management, but also have an increased risk of lung cancer due to dysregulation of genes, immune system and microenvironment in the pathomechanism. Therefore, the development of safer and more effective treatments for COPD is particularly important. Optimizing treatment regimens can improve patients’ quality of life while effectively reducing the risk of lung cancer, thus improving the overall health of COPD patients while reducing the potential threat of cancer (7).

COPD is categorized as “cough” and “lung swelling” in Chinese medicine, and its main symptoms include cough, phlegm, wheezing and chest tightness. Plants are rich in bioactive substances, such as flavonoids, polyphenols and terpenoids, which have important applications in the prevention and treatment of various diseases through a variety of biological mechanisms, such as antioxidant, anti-inflammatory, and immunomodulatory. Some studies have shown the efficacy of *Ardisia japonica* (Thunb.) Blume (Zijinniu) and *Lespedeza cuneata* G. Don (Tiesaozhou), in COPD treatment (8, 9). In addition, Zijinniu has shown effects of modulating inflammatory responses and improving blood rheology, which may be one of the potential mechanisms for its treatment of blood stasis (10–12), whereas the process of pulmonary vascular injury and obstruction of blood flow in patients with COPD is similar to the manifestation of blood stasis, where abnormalities in blood flow lead to a decrease in the efficiency of gas exchange, which in turn affects the patient’s overall health status (13). Zijinniu is effective in chronic bronchitis and inflammation of the upper respiratory tract. Zijinniu and Tiesaozhou are traditional herbs of the Miao people of Guizhou with remarkable curative effects, especially in relieving cough and resolving phlegm (14). Zijinniu is a small evergreen shrub of the genus Purple golden ox in the family Primulaceae, rich in a variety of bioactive components, including flavonoids, sugars and organic acids. Its main active ingredient is the dwarf camptothecin (petitgrain), which is considered to be the key ingredient in its cough-relieving and asthma-relieving effects (15, 16). Tiesaozhou is a small shrub of the genus Tiesaozhou in the family Leguminosae, rich in flavonoids, polyphenols and saponins antioxidant components, which are able to scavenge free radicals, reduce oxidative stress and inhibit the release of inflammatory mediators, which in turn reduces inflammation in the lungs and aids in the prevention and treatment of COPD (17–19). Despite the positive effects of Zijinniu and Tiesaozhou in the treatment of COPD, the specific mechanism of action is not yet completely clear, and further research is still needed to explore.

Network pharmacology has emerged as a powerful approach to predict the therapeutic mechanisms of TCM. By screening active ingredients, identifying targets, constructing disease target databases, and conducting enrichment analyses, network pharmacology offers valuable insights for addressing research challenges related to TCM (20–23). Purple golden cow [*Ardisia japonica* (Thunb.) Blume] and iron broom (*Lespedeza cuneata*) are traditional herbs of the Miao people of Guizhou with remarkable curative effects, especially in relieving cough and resolving phlegm. Purple golden ox is a small evergreen shrub of the genus Purple golden ox in the family Primulaceae, rich in a variety of bioactive components, including flavonoids, sugars and organic acids. Its main active ingredient is the dwarf camptothecin (petitgrain), which is considered to be the key ingredient in its cough-relieving and asthma-relieving effects. Through synergistic multi-compound network pharmacology and drug repurposing, precise and effective therapeutic interventions can be achieved, bypassing the need for extensive drug development and accelerating clinical application (24). Thus, utilizing network pharmacology to analyze the mechanisms of action, predict drug targets, and evaluate the efficacy of Zijinniu and Tiesaozhou in COPD represents a significant advancement in COPD treatment strategies.

This study leverages data from the GEO public database, along with the active ingredients of Zijinniu and Tiesaozhou obtained from the Herbal Ingredients’ Targets database and literature sources, to predict target genes. By combining network pharmacology and bioinformatics, this study explores the potential targets of Zijinniu and Tiesaozhou in COPD treatment, further investigating the molecular regulatory mechanisms of key genes involved in COPD. These findings provide valuable insights into the mechanisms of action of Zijinniu and Tiesaozhou, offering new references for their use in COPD therapy.

## 2 Materials and methods

### 2.1 Data source

Three transcriptomic datasets, GSE124180, GSE248493 and GSE42057, sourced from the Gene Expression Omnibus (GEO),^1^ were included in this study. Specifically, GSE124180 contained 6 COPD and 15 healthy control peripheral blood samples, based on the GPL16791 platform, GSE248493 included 25 COPD and 12 health control peripheral blood samples relied on GPL18573 platform, while GSE42057 consisted of 94 COPD and 42 healthy control peripheral blood samples, utilizing the GPL570 platform. Additionally, the active ingredients of Zijinniu were sourced from the Herbal Ingredients’ Targets database (HERB),^2^ and the active ingredients of Tiesaozhou were obtained from previously published literature (25).

### 2.2 Differential expression analysis

Differential expression analysis was conducted on the GSE124180 dataset using the “DESeq2” package (v 3.4.1) (26) to identify differentially expressed genes (DEGs) between COPD and control samples. The criteria for significance were |log_2_Fold Change (FC)| > 0.5 and *P* < 0.05. DEG visualization was achieved using the “ggplot2” (v 3.4.1) (27) and “ComplexHeatmap” (v 2.14.0) (28) packages, which produced a volcano plot and heatmap, respectively.

### 2.3 Construction of Chinese medicinal herb-active ingredient-target genes network

To predict potential target genes for the active ingredients of Zijinniu and Tiesaozhou, the HERB and PubChem databases were utilized, followed by target gene prediction using the SwissTargetPrediction tool. Specifically, target genes for the active ingredients of Zijinniu (target genes 1) and Tiesaozhou (target genes 2) were predicted. Cytoscape software (v 3.7.2) (29) was then used to construct and visualize the network linking Chinese medicinal herbs, active ingredients, and target genes.

### 2.4 Function analysis of candidate genes

Candidate genes were identified by overlapping DEGs with target genes 1 and target genes 2 using the “VennDiagram” package (v 1.7.1) (30). To explore the potential biological processes and signaling pathways associated with these candidate genes, gene ontology (GO) and Kyoto Encyclopedia of Genes and Genomes (KEGG) enrichment analyses were performed using the “ClusterProfiler” package (v 4.7.1.3) (31), applying a significance threshold of *P* < 0.05. GO analysis included cellular components (CC), biological processes (BP), and molecular functions (MF).

### 2.5 Identification of the key genes

Gene expression analyses of the candidate genes in both GSE124180 and GSE42057 datasets were performed using the Wilcoxon test (COPD vs. control). Genes exhibiting significant differences (*P* < 0.05) and consistent expression patterns across both datasets were selected as key genes for further analysis. The results from both datasets were visualized using the “ggplot2” package.

### 2.6 Establishment and evaluation of the nomogram

To assess the risk of COPD development, a nomogram was constructed using the “rms” package (v 6.5.0) (32), based on the identified key genes and samples from the GSE124180 dataset. A calibration curve (threshold *P* > 0.05) was generated to evaluate the nomogram’s accuracy, where a slope closer to 1 indicated improved predictive accuracy. Receiver operating characteristic (ROC) curves were plotted using the “pROC” package (v 1.18.5) (33), with a threshold of area under the curve (AUC) > 0.7 to evaluate the nomogram’s predictive performance. Then, based on the dataset GSE248493, a nomogram was constructed, and the model performance was evaluated using calibration curve and ROC curve.

### 2.7 Construction of key genes-organ/tissue network

To investigate the specific distribution of key genes across various tissues and organs, mRNA expression data for each key gene were obtained from the Biological Gene Portal System database (BioGPS).^3^ Relevant organs and tissues with mRNA expression levels above the overall mean for each key gene were extracted. These data were used to construct a key genes-organ/tissue network, visualizing the localization of key genes across different organs and tissues, which was then displayed using Cytoscape.

### 2.8 Correlation analysis and function analysis of key genes

Spearman analysis was performed on the GSE124180 dataset using the “cor” function in R to investigate the relationships among key genes. A threshold of |Cor| > 0.3 and *P* < 0.05 was applied to determine the strength and significance of associations between key genes.

GeneMANIA^4^ was employed to identify functionally related genes, and a gene-gene interaction (GGI) network was constructed. To explore the biological functions of the key genes, the “c2.cp.kegg.v2023.1.Hs.symbols.gmt” file was downloaded from the gene set enrichment analysis (GSEA) website^5^ as the background gene set. GSEA analysis was then conducted using the “clusterProfiler” package based on the GSE124180 dataset, with a significance threshold of *P* < 0.05.

### 2.9 Immune factor analysis

To examine the correlation between key genes and immune factors, data on related immune factors, including chemotactic factors, immunosuppressants, immunostimulants, chemokine receptors, and major histocompatibility complex (MHC), were downloaded from the Tumor and Immune System Interaction Database (TISIDB).^6^ Spearman analysis was subsequently performed to explore associations between key genes and these immune factors, applying a threshold of |Cor| > 0.3 and *P* < 0.05.

### 2.10 Regulatory network analysis

To elucidate the molecular regulatory mechanisms of key genes, transcription factors (TFs) targeting these genes were predicted using the Just Another Gibbs Sampling (JASPAR)^7^ database *via* the NetworkAnalyst platform. A TF-mRNA network was subsequently constructed and visualized using Cytoscape.

The miRTarBase^8^ database, accessed through the NetworkAnalyst platform, was utilized to predict microRNAs (miRNAs) associated with the key genes. Following this, the miRNet database^9^ was used to predict long non-coding RNAs (lncRNAs) targeting the identified miRNAs. These relationships were integrated to construct and visualize a comprehensive lncRNAs-miRNAs-mRNAs network using Cytoscape.

### 2.11 Diseases prediction and molecular docking analysis

To examine the relationship between key genes and diseases, the Comparative Toxicogenomics Database (CTD)^10^ was utilized, and inference scores were calculated. The top 10 diseases with the highest inference scores were then selected to construct a network linking key genes to diseases.

Molecular docking was performed to assess the binding affinities between the key genes and active ingredients. Active ingredients with the highest Oral Bioavailability (OB) scores in Zijinniu and Tiesaozhou were selected for docking analysis. The 3D structures of the key genes (receptors) were retrieved from the Research Collaboratory for Structural Bioinformatics Protein Data Bank (RCSB PDB),^11^ while the 3D structures of the key active ingredients (ligands) were downloaded from the PubChem database.^12^ Molecular docking was conducted using the cb-dock website,^13^ and the total docking score was calculated. A binding energy of < −7.0 kcal/mol indicated a strong binding affinity.

### 2.12 Metabolic pathways analysis

In the GSE124180 dataset, the potential impact of body metabolism on COPD was investigated. Metabolic pathway data were sourced from the Molecular Signatures Database (MSigDB).^14^ Spearman correlation analysis was performed to assess the relationship between key genes and metabolic pathways, with a threshold of |cor| > 0.3 and *P* < 0.05 used to determine significant correlations.

### 2.13 Expression validation of key genes

The expression of key genes was validated through RT-qPCR. A total of 10 blood samples (5 normal and 5 COPD) were obtained from the clinic at The Second Affiliated Hospital of Guizhou University of Traditional Chinese Medicine. Informed consent was obtained from all participants, and the study was approved by the hospital’s ethics committee (approval number: 2W20240601).

Total RNA was extracted from the samples using TRIzol reagent (Ambion, USA) according to the manufacturer’s protocol. RNA concentration was assessed using the NanoPhotometer N50. cDNA synthesis was performed using the SureScript-First-strand-cDNA-synthesis-kit, and reverse transcription was carried out with the S1000™ Thermal Cycler (Bio-Rad, USA). Primer sequences are listed in Supplementary Table 1. qPCR was conducted using the CFX Connect Real-time Quantitative Fluorescence PCR Instrument (Bio-Rad, USA) with the following protocol: pre-denaturation at 95°C for 1 min, denaturation at 95°C for 20 s, annealing at 55°C for 20 s, extension at 72°C for 30 s, for a total of 40 cycles. Relative mRNA quantification was calculated using the 2^ΔΔCT^ method.

### 2.14 Statistical analysis

All analyses were performed in R software (v 4.2.3). Differences between groups were evaluated using the Wilcoxon test, and *P* < 0.05 was considered statistically significant.

## 3 Results

### 3.1 The function and enriched pathways of six candidate genes were mined

**FIGURE 1:**
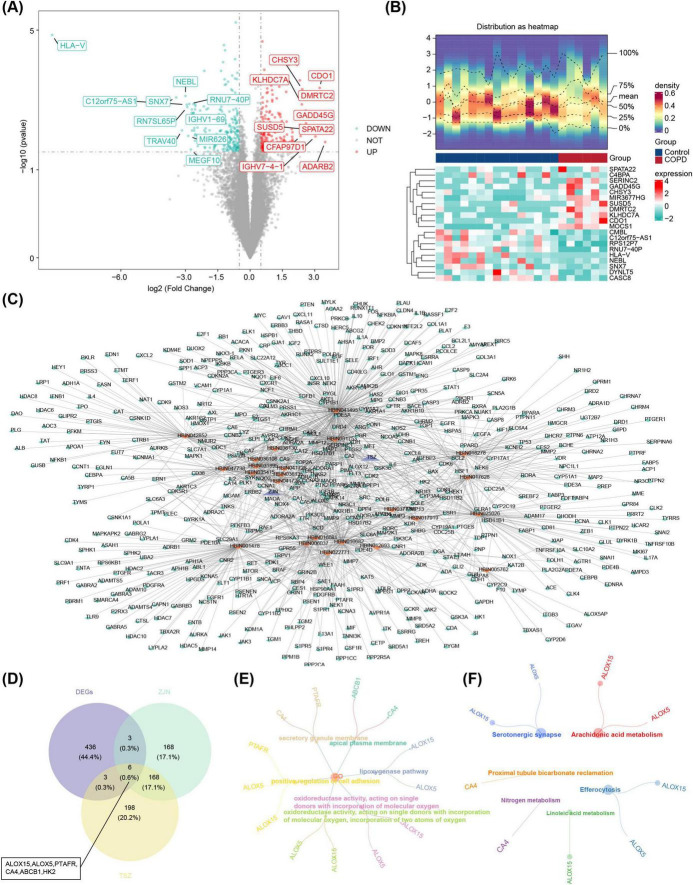
Identification and functional enrichment of candidate genes. **(A)** Differential expression volcano plot. The horizontal axis represents the fold change in gene expression, and the vertical axis shows the –log10 of the *P*-value. Red dots indicate upregulated genes, green dots indicate downregulated genes, and grey dots represent non-differentiated genes. **(B)** Heatmap of differentially expressed genes. The image consists of two parts: the upper section displays the heatmap of gene expression density, with lines representing the five quartiles and the mean; the lower section shows the heatmap of differential gene expression across samples, where each row represents a gene (top 10 genes with the most significant up- and downregulation), and each column represents a sample. Red areas correspond to disease samples, while blue areas indicate control samples. **(C)** Herbal medicine-active ingredient-target gene network. Purple represents herbal medicine: Zijinniu, Tiesaozhou. Orange represents active ingredients, Zijinniu: 15, Tiesaozhou:8. And green represents active ingredient’s potential target genes. The more lines around the orange node, the more potential target genes predicted by the active ingredients. **(D)** The identification of six candidate genes: ALOX15, ALOX5, PTAFR, CA4, ABCB1, HK2. **(E,F)** GO and KEGG enrichment analyses. HK2 did not show significant results in the GO and KEGG enrichment analyses. **(E)** GO enrichment map of candidate gene. **(F)** KEGG enrichment of candidate genes.

In the GSE124180 dataset, 448 DEGs were identified, comprising 251 up-regulated and 197 down-regulated genes. The DEG results were visualized in both a volcano plot and a heatmap, with the top 10 up- and down-regulated DEGs, ranked by log_2_FC, highlighted (Figures 1A, B).

Additionally, 15 active ingredients for Zijinniu and 8 for Tiesaozhou were identified, along with 345 target genes for Zijinniu and 375 target genes for Tiesaozhou. These findings facilitated the construction of a network encompassing Chinese medicinal herbs, active ingredients, and target genes, which consisted of 571 nodes and 1,362 edges. Notable network connections included Zijinniu-HBIN041726-NQO2 and Tiesaozhou-HBIN031753-DRQ4 (Figure 1C). Six candidate genes were identified through overlap of the 448 DEGs, 345 target genes for Zijinniu, and 375 target genes for Tiesaozhou (Figure 1D). Functional enrichment analysis revealed that these 6 candidate genes were significantly enriched in 334 GO terms (278 BPs, 24 CCs, and 32 MFs) and 19 KEGG pathways. Key GO terms included “lipoxygenase pathway,” “positive regulation of cell adhesion,” and “apical plasma membrane” (Figure 1E). The KEGG pathways included “efferocytosis,” “arachidonic acid metabolism,” and “serotonergic synapse” (Figure 1F).

### 3.2 HK2 and PTAFR were identified as key genes

**FIGURE 2:**
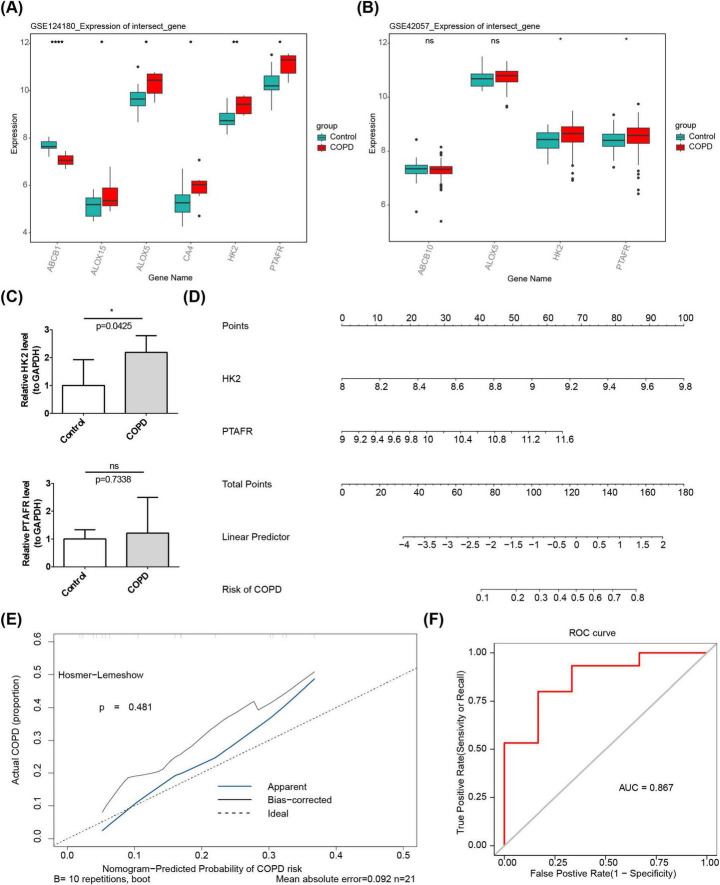
Expression of key genes HK2 and PTAFR in COPD. **(A)** Differential expression of candidate targets between disease and control groups in the training set. **(B)** Differential expression of candidate targets between disease and control groups in the validation set. **(C)** RT-qPCR analysis. **(D)** Nomination plot based on key gene constructs. (1) The variable names on the left represent the factors, with each line corresponding to a variable. The length of the line segment reflects the magnitude of its contribution to the disease. The scale indicates the possible range of values for each variable. (2) Score: Individual score corresponds to the score for each variable at different values, while total score represents the sum of individual scores. (3) Linear predictor: The linear predictive value for the variables. (4) Predictive probability: Indicates the likelihood of disease development. **(E)** Calibration curve. The horizontal axis represents the predicted risk of disease based on the column-line graph model, and the vertical axis represents the actual disease occurrence probability. “Ideal” denotes the ideal calibration curve, “Apparent line” represents internal correction, and “Bias-corrected line” represents external correction. **(F)** ROC curve. The horizontal axis represents the false-positive rate (1-specificity), and the vertical axis represents the true-positive rate (sensitivity). The AUC value reflects the area under the ROC curve. *P < 0.05, **P < 0.01, ****P < 0.0001.

Gene expression analysis of datasets GSE124180 and GSE42057 revealed significant increases in the expression levels of HK2 and PTAFR in COPD samples (*P* < 0.05) (Figures 2A, B). Consequently, HK2 and PTAFR were identified as key genes for further investigation. RT-qPCR analysis demonstrated that the expression of HK2 (*P* = 0.0425) was significantly elevated in COPD samples, while no significant difference was observed for PTAFR (*P* = 0.7338) between COPD and control samples (Figure 2C).

Using these key genes, a nomogram was constructed, where higher total points correlated with a higher risk of COPD (Figure 2D). The calibration curve yielded a *P*-value of 0.481, indicating strong predictive performance (Figure 2E). Furthermore, ROC analysis demonstrated the nomogram’s high efficacy association with COPD risk, with an AUC value of 0.867 (Figure 2F). Then, we performed external validation of the model using the dataset GSE248493. The results showed a calibration curve *p* = 0.411 and AUC = 0.831, indicating that the model had good accuracy in the external dataset (Supplementary Figure 1).

### 3.3 Investigating the biological functions and signaling pathways associated with HK2 and PTAFR

**FIGURE 3:**
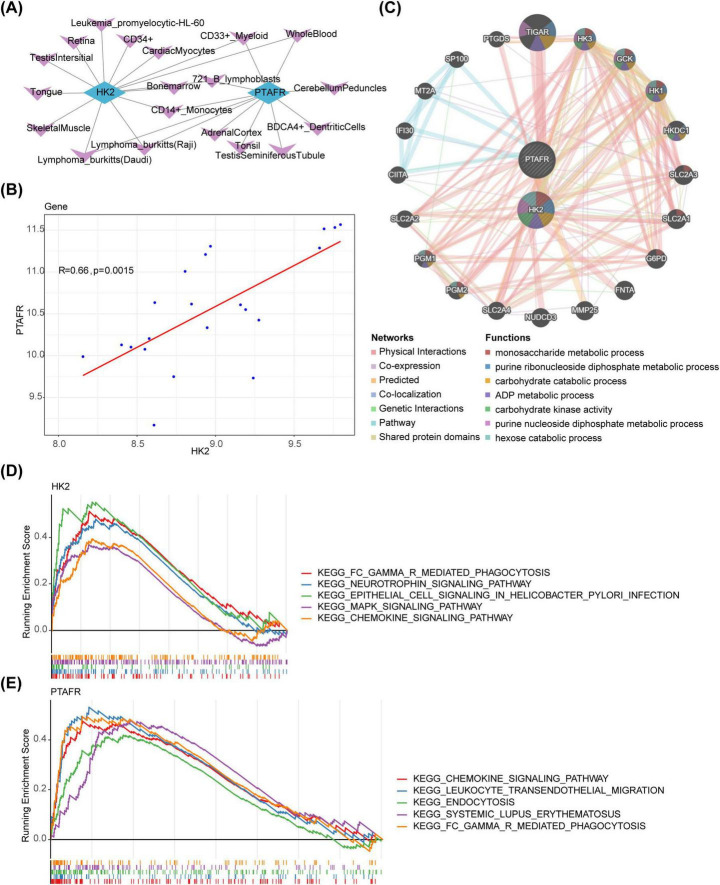
Biological functions and signaling pathways of HK2 and PTAFR in disease. **(A)** Key target-organ localization network diagram. Blue represents key targets, and purple represents organs. **(B)** Key target correlation scatter plot. **(C)** Analysis of key targets and their co-expressed genes by GeneMANIA. The center circle represents the two key targets, and the outer circle displays co-expressed genes associated with the hub genes. **(D)** Significantly enriched pathways for HK2. In this figure, the vertical axis represents the enrichment scores (ES). A positive ES indicates that a functional gene is enriched at the beginning of the sorted sequence, implying positive enrichment, while a negative ES suggests that the functional gene is enriched at the end of the sequence, indicating negative enrichment. The horizontal axis represents genes, with each small vertical line corresponding to an individual gene. **(E)** Significantly enriched pathway of PTAFR. In this figure, the vertical axis represents the ES. A positive ES indicates that a functional gene is enriched at the beginning of the sequence, reflecting positive enrichment, while a negative ES indicates that the gene is enriched at the end of the sequence, reflecting negative enrichment. The horizontal axis represents genes, with each small vertical line corresponding to an individual gene.

To delineate the organ and tissue distribution of key genes, a key genes-organ/tissue network was constructed, revealing that 14 and 11 organs and tissues were associated with HK2 and PTAFR, respectively. Six tissues were common to both genes: 721 B lymphoblasts, CD14+ monocytes, CD33+ myeloid cells, Wholeblood, Lymphoma Burkitts (Raji), and Lymphoma Burkitts (Daudi) (Figure 3A). Additionally, correlation analysis revealed a significant positive correlation between HK2 and PTAFR (*r* = 0.66, *P* = 0.0015) (Figure 3B). Using these key genes, 20 functionally related genes were predicted, and the shared biological functions they contribute to were identified, including “monosaccharide metabolic process” and “purine ribonucleoside diphosphate metabolic process” (Figure 3C). Moreover, GSEA uncovered distinct pathways enriched with HK2 and PTAFR (*P* < 0.05). Specifically, HK2 was significantly enriched in the “Neurotrophin signaling pathway,” “Epithelial cell signaling in Helicobacter pylori infection,” and “MAPK signaling pathway,” while PTAFR was primarily involved in “Leukocyte transendothelial migration,” “Endocytosis,” and “Systemic lupus erythematosus” pathways. Notably, both HK2 and PTAFR co-enriched in the “Chemokine signaling pathway” and “FC gamma R-mediated phagocytosis” pathways (Figures 3D, E).

### 3.4 HK2 and PTAFR played intricate roles in modulating various aspects of the immune response

**FIGURE 4:**
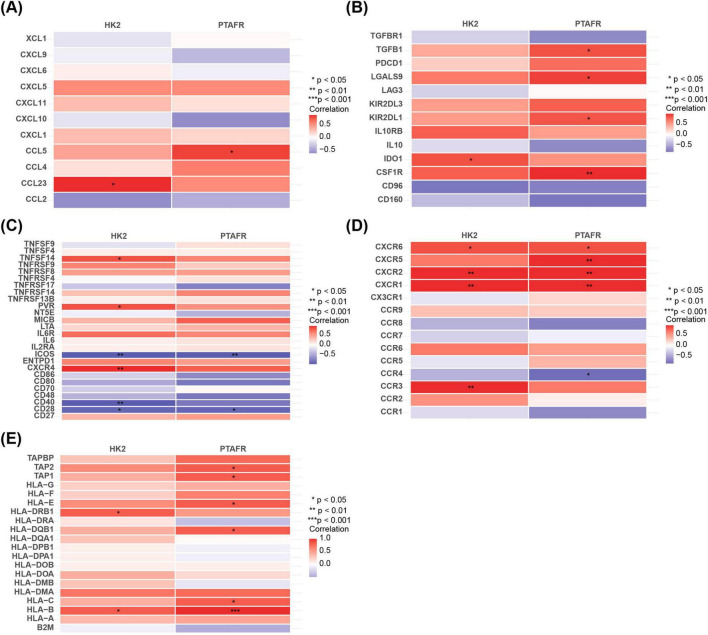
Relationship between immunofactor analysis and HK2 and PTAFR. **(A)** Correlation of chemokines with key targets. **(B)** Correlation of immunosuppressants with key targets. **(C)** Correlation of immunostimulatory factors with key targets. **(D)** Correlation of chemokine receptors with key targets. **(E)** Correlation of major histocompatibility complexes with key targets.

Correlation analysis between key genes and immune-related factors revealed notable associations. In chemotactic factors, HK2 showed a significantly positive correlation with CCL23, while PTAFR was positively correlated with CCL5 (*P* < 0.05) (Figure 4A). Regarding immunosuppressants, HK2 demonstrated a positive correlation with IDO1, whereas PTAFR was positively correlated with CSF1R (*P* < 0.05) (Figure 4B). In immunostimulants, HK2 exhibited a significant positive correlation with CXCR4 and a negative correlation with ICOS, whereas PTAFR showed a notable negative correlation with ICOS (*P* < 0.05) (Figure 4C). For chemokine receptors, HK2 displayed significant positive correlations with CXCR1, CXCR2, and CXCR3, while PTAFR was positively correlated with CXCR1 and CXCR2 but negatively correlated with CCR4 (*P* < 0.05) (Figure 4D). Finally, with respect to MHC molecules, HK2 showed significant positive correlations with HLA-DRB1 and HLA-B, while PTAFR had the strongest positive correlation with HLA-B and TAP1 (*P* < 0.05) (Figure 4E). In conclusion, the correlation analysis highlighted the intricate associations between HK2 and PTAFR and various immune-related factors, underscoring their potential roles in modulating diverse aspects of the immune response.

### 3.5 Unraveling regulatory networks of HK2 and PTAFR

**FIGURE 5:**
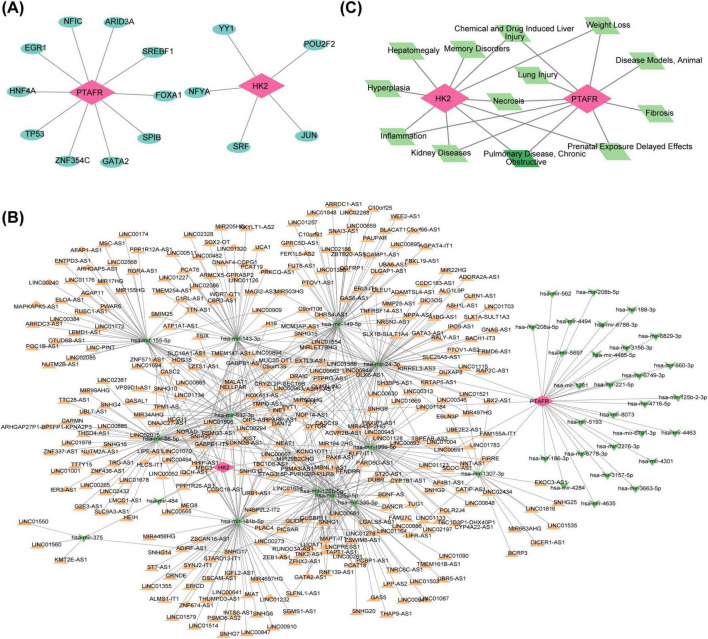
Regulatory relationships between key targets and transcription factors and related molecular regulatory networks. **(A)** TF-mRNA network diagram. Red nodes represent mRNAs (key genes), and blue nodes represent transcription factors. **(B)** Construction of the lncRNAs-miRNAs-mRNAs regulatory network for key targets. Red nodes represent mRNAs, green nodes represent miRNAs, and orange nodes represent lncRNAs. **(C)** Key target-COPD-TOP10 related disease regulatory network. Red nodes represent mRNAs, green nodes represent COPD, and light green nodes represent the correlative top 10 diseases.

Five TFs targeting HK2 and ten TFs targeting PTAFR were predicted. A TF-mRNA network was subsequently constructed, comprising 17 nodes and 16 edges, with key relationships such as ARID3A-PTAFR and YY1-HK2 (Figure 5A). Additionally, 42 miRNAs (9 targeting HK2 and 33 targeting PTAFR) associated with the two key genes were predicted. Furthermore, 589 miRNA-lncRNA relationships were retrieved from the miRNet database, leading to the construction of an lncRNA-miRNA-mRNA interaction network (Figure 5B), featuring interactions such as LIFR-AS1-“hsa-mir-1307-3p”-PTAFR and MIR99AHG-“hsa-mir-98-5p”-HK2. Disease association analysis, performed via the CTD database, identified the top 10 diseases (ranked by inference scores) linked to HK2 and PTAFR. Among these, “Chemical and drug-induced liver injury,” “Weight loss,” “Pulmonary disease, Chronic Obstructive,” “Prenatal exposure delayed effects,” and “Necrosis” were found to be associated with both HK2 and PTAFR (Figure 5C).

### 3.6 The quercetin and daucosterol were potential active ingredients for treating COPD

**FIGURE 6:**
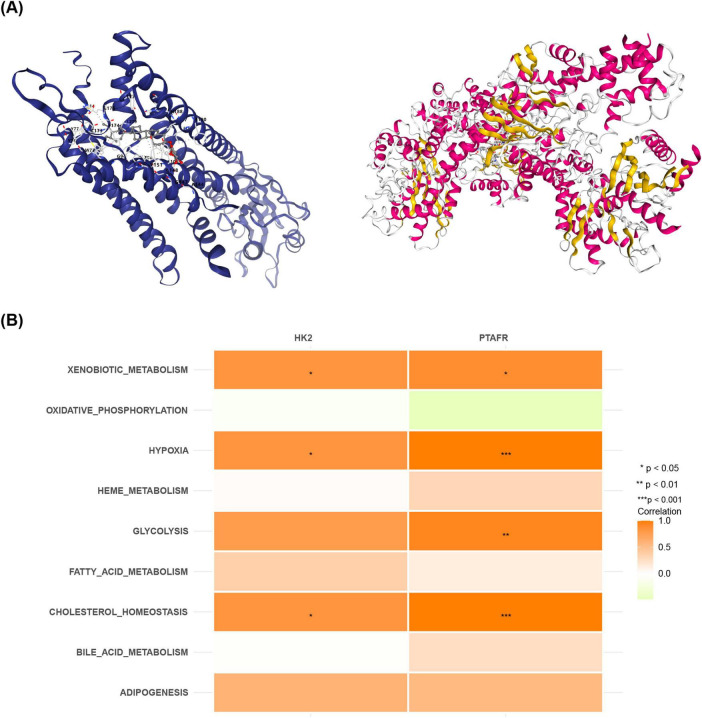
Relationships between pharmacologically active components and target genes in Zijinniu and Tiesaozhou. **(A)** HK2-quercetin molecular docking. **(B)** PTAFR-daucosterol molecular docking.

Based on OB scores, quercetin (OB = 46.433) and daucosterol (OB = 20.631) were selected for molecular docking analysis (Supplementary Table 2). The PDB code of HK2 is 2nzt, and the PDB code of PTAFR is 5zkp. Quercetin demonstrated a binding energy of −8.2 kcal/mol with HK2, while daucosterol showed a binding energy of −8.4 kcal/mol with PTAFR (Figure 6A). These results suggest that both quercetin and daucosterol exhibit strong binding affinities with the key genes. Moreover, metabolic pathway analysis revealed that both HK2 and PTAFR were significantly positively correlated with “Xenobiotic metabolism,” “Hypoxia,” and “Cholesterol homeostasis” (*P* < 0.05) (Figure 6B). Additionally, PTAFR showed a notable positive correlation with “Glycolysis” (*P* < 0.05) (Figure 6B).

## 4 Discussion

COPD is categorized as “cough” and “lung distension” in TCM. Purple golden ox and iron broom refer to a traditional medicinal pair, these two herbs are analyzed together because they are similar in nature and both belong to the lung meridian in traditional Chinese medicine (TCM), and their effects complement each other, making them suitable for pairing and use, and when used together, they can synergistically enhance the effect of resolving phlegm and relieving coughs, and they have been used for a long time to treat respiratory disorders in the theory of traditional Chinese medicine (TCM) (34). Modern pharmacological research has demonstrated that the active ingredients of Tiesaozhou exhibit various therapeutic effects, including asthma alleviation, antifungal, lipid-lowering, and antitumor properties, making it effective in the treatment of chronic bronchitis. Additionally, both Zijinniu and Tiesaozhou can counteract several major pathogenic inflammatory factors in COPD through specific pathways, inhibit the proliferation of airway fibrous tissues in COPD rat models, and slow down the progression of the disease. In this study, network pharmacology was used to analyze the targets of the active ingredients in Zijinniu and Tiesaozhou, confirming their potential to delay COPD progression, particularly pulmonary distension, through multi-target and multi-gene mechanisms.

GO and KEGG enrichment analyses of candidate genes identified HK2 and PTAFR as key targets. HK2, an isoform of hexokinase (HK), plays a pivotal role in glucose metabolism. It is highly expressed in muscle, heart, and various types of cancers, with enriched expression in mouse and human microglial cells (35, 36). Ablation of HK2 significantly promotes microglial activation and phagocytic activity, along with increased levels of inflammatory markers such as tumor necrosis factor, interleukin (IL)-6, IL-1B, and type I interferon response molecules (37). This suggests that HK2 enhances the inflammatory response, thereby contributing to disease progression. Furthermore, HK2 overexpression in tumor cells has been shown to promote the proliferation of non-small cell lung cancer cells and tumor growth, highlighting its role in cancer development (38). In this study, the expression of HR2 in the COPD group was significantly higher than in the normal control group, suggesting that HK2 may enhance the inflammatory response and contribute to the progression of COPD. Regarding platelet-activating factor receptor (PTAFR), platelet-activating factor is a multifunctional phospholipid that exerts pro-inflammatory, pro-coagulant, and angiogenic effects on blood vessels (39). PTAFR is present in various intracellular locations, including the nucleus, and is implicated in a wide range of cancers and disease processes. Respiratory pathogens, such as viruses and bacteria, utilize PTAFR to interact with human cells and initiate infections (40). PTAFR has also been identified as a major driver of lung inflammation during COVID-19 viral infections, with inhibition of PTAFR shown to protect against the severe consequences and lung damage caused by pathogens (41). These findings suggest that PTAFR plays a significant role in lung inflammation. This study is the first to highlight the potential involvement of these two genes in the development of COPD. Our results demonstrate that the expression levels of HK2 and PTAFR were significantly elevated in COPD, supporting the hypothesis that both genes may contribute to the disease process. Consequently, down-regulating the expression of HK2 and PTAFR could prevent or delay COPD progression. Furthermore, a nomogram model was constructed based on these two key genes, which exhibited predictive value as assessed by calibration and ROC curves.

Additionally, immunofactor analyses revealed significant correlations between HK2 and PTAFR and various chemokines (e.g., CCL23, CCL5), chemokine receptors (e.g., CXCR1, CXCR2), immunosuppressants (e.g., IDO1, CSF1R), immunostimulants (e.g., ICOS, CD28), and HLA-B. The expression of chemokines and their receptors is associated with several inflammatory diseases, as they recruit leukocytes and promote the release of inflammatory mediators. These molecules are involved in homeostasis, development, angiogenesis, tumor-associated inflammation, immunity, tumor growth, metastasis, and autoimmune diseases. Chemokines also play a critical role in cellular fibrosis and senescence. Additionally, CCL5 expression is significantly increased in senescent fibroblasts (42). Previous studies have demonstrated that the levels of CCL5, CXCL8, CXCL12, and CX3CL1 correlate with lung function, particularly MMEF, with CXCL8 and CX3CL1 exhibiting the strongest associations with MMP-9 and MMP-12. These findings suggest that chemokines may play a direct or indirect role in airway remodeling (43). Furthermore, targeting chemokine receptors, including CXCR1, CXCR2, and CD8 on neutrophils, as well as CXCR3 on CD8^+^ T cells, could prevent the recruitment of healing cells to inflamed lungs (44) and may offer a potential therapeutic strategy for COPD (45). However, ICOS, an immunostimulatory factor, is crucial in humans, where its deficiency leads to common variable immunodeficiency (46), predisposing individuals to frequent bacterial infections in the respiratory and digestive tracts (47). COPD is recognized as an immunodeficiency disorder, and defects in the immunostimulatory factor ICOS have been linked to its development. The ICOS-mediated signaling pathway regulates T lymphocytes and controls their selective migration into inflamed peripheral tissues. Additionally, the persistence of bacteria in the airways of COPD is attributed to the mediation of this ICOS signaling pathway (48), suggesting that ICOS deficiency contributes to COPD pathogenesis. In the present study, a significant negative correlation between HK2, PTAFR, and ICOS was identified, suggesting that patients with COPD exhibit reduced or defective ICOS expression. Additionally, research on SARS-CoV-2 infection has indicated that enhancing the expression of human leukocyte antigen-B (HLA-B) may facilitate viral clearance (49), implying that HLA-B could play a role in inflammation modulation. In the present study, a significant positive correlation between HK2, PTAFR, and HLA-B was observed, with higher expression levels of both HK2 and PTAFR in patients with COPD corresponding to increased HLA-B expression. These findings highlight the association between chemokines, chemokine receptors, and immunostimulatory factors in COPD. Furthermore, our results confirmed that CCL5 and ICOS are positively correlated with HK2 and PTAFR, both of which are highly expressed in patients with COPD. Thus, chemokines, chemokine receptors, immunostimulatory factors, HLA-B, HR2, and PTAFR collectively mediate COPD development.

Based on the molecular docking simulation results, we hypothesized that the active ingredients of Zijinniu and Tiesaozhou might bind to and inhibit HK2 and PTAFR, but this conclusion needs to be confirmed by further experimental validation. Quercetin (3,3′,4′,5,7-pentahydroxyflavone), a dietary flavonoid present in various plants, is the active component in Zijinniu officinalis. At the molecular level, quercetin functions as an antioxidant by scavenging reactive oxygen species (50), inhibits a range of kinases with anti-inflammatory effects (51), and reduces basal cell hyperplasia, squamous epithelial chemotaxis, and cupping chemotaxis (52, 53). In bronchial epithelial cells from patients with COPD, quercetin significantly upregulates the expression of HOXB2 and ELF3, facilitating bronchial epithelial polarization and repair, thus promoting normal cellular differentiation and reducing the expression of TGF-β and IL-8 (54). Furthermore, quercetin holds potential in controlling and preventing bacterial infections in the lower respiratory tract in COPD, while potentially enhancing the effects of β-agonists, M-receptor antagonists, corticosteroids, roflumilast, antibiotics, and N-acetylcysteine through bronchodilatory, anti-inflammatory, antibacterial, and antiviral actions, respectively (55–57). Daucosterol, the active ingredient in Tiesaozhou, has been shown to induce oxidative stress-mediated apoptosis in various cancer cell lines (58). Since oxidative stress is a key mechanism underlying COPD development, daucosterol may offer protection in acute lung injury models by inhibiting p38 through regulation of NLRP3 inflammasome activation (59, 60). This suggests that quercetin and daucosterol may influence cell proliferation, pointing to their therapeutic potential in COPD.

In this study, network pharmacology and bioinformatics analyses identified two key COPD-related genes, HK2 and PTAFR, through differential expression analysis and gene screening. Using these approaches, the active ingredients quercetin and daucosterol were docked with HK2 and PTAFR, revealing potential mechanisms of action. These findings may provide novel insights into the clinical diagnosis and treatment of COPD. However, Our study still has some limitations, the insufficient sample size of RT-qPCR may lead to insufficient statistical efficacy, which makes the experimental results unable to accurately reflect the real biological effects, and molecular docking only provides a theoretical model, and the calculation of affinity is only a preliminary speculation, which can not directly reflect the actual biological activity, so we plan to increase the sample size to enhance the statistical efficacy of the RT- Therefore, we plan to increase the sample size in future studies to enhance the statistical efficacy of RT-qPCR experiments, and at the same time conduct multiple independent experiments for validation and cross-validation of data in combination with other research methods, so as to improve the reliability and generalizability of the results.

## Data Availability

Publicly available datasets were analyzed for this study. These data can be found here: The dataset analyzed for this study can be found in GEO (http://www.ncbi.nlm.nih.gov/geo/), GSE124180, GSE248493 and GSE42057, and the Herbal Ingredient Targets Database (HERB), http://herb.ac.cn/.

## Footnotes

1
https://www.ncbi.nlm.nih.gov/gds

2
http://herb.ac.cn/

3
https://biogps.org

4
https://genemania.org/

5
https://www.gsea-msigdb.org/gsea/msigdb/index.jsp

6
http://cis.hku.hk/TISIDB/

7
https://jaspar.genereg.net/

8
http://mirtarbase.mbc.nctu.edu.tw/

9
https://www.mirnet.ca/miRNet/home.xhtml

10
http://ctdbase.org

11
https://www.rcsb.org/

12
https://pubchem.ncbi.nlm.nih.gov/

13
https://cadd.labshare.cn/cb-dock/php/blinddock.php

14
https://www.gsea-msigdb.org/gsea/msigdb

## References

[1] Christenson S Smith B Bafadhel M Putcha N. Chronic obstructive pulmonary disease. *Lancet.* (2022). 399:2227–42. 10.1016/S0140-6736(22)00470-6 35533707

[2] Yang I Jenkins C Salvi S. Chronic obstructive pulmonary disease in never-smokers: Risk factors, pathogenesis, and implications for prevention and treatment. *Lancet Respir Med.* (2022) 10:497–511. 10.1016/S2213-2600(21)00506-3 35427530

[3] Calverley P Walker P. Contemporary Concise Review 2022: Chronic obstructive pulmonary disease. *Respirology.* (2023) 28:428–36. 10.1111/resp.14489 36922031

[4] Ritchie A Wedzicha J. Definition, causes, pathogenesis, and consequences of chronic obstructive pulmonary disease exacerbations. *Clin Chest Med.* (2020) 41:421–38. 10.1016/j.ccm.2020.06.007 32800196 PMC7423341

[5] Yousuf A Mohammed S Carr L Yavari Ramsheh M Micieli C Mistry V Astegolimab, an anti-ST2, in chronic obstructive pulmonary disease (COPD-ST2OP): A phase 2a, placebo-controlled trial. *Lancet Respir Med.* (2022) 10:469–77. 10.1016/S2213-2600(21)00556-7 35339234

[6] Nathan S Argula R Trivieri M Aziz S Gay E Medarov B Inhaled treprostinil in pulmonary hypertension associated with COPD: PERFECT study results. *Eur Respir J.* (2024) 63:2400172. 10.1183/13993003.00172-2024 38811045 PMC11154754

[7] Forder A Zhuang R Souza V Brockley L Pewarchuk M Telkar N Mechanisms contributing to the comorbidity of COPD and lung cancer. *Int J Mol Sci.* (2023) 24:2859. 10.3390/ijms24032859 36769181 PMC9918127

[8] Liu M Xun Z Wang M Li C. Intervention study on airway DCN and Smad7 in COPD rats by Zijinxu and Iron broom. *Chin Med.* (2019) 8:314–24. 10.12677/tcm.2019.85052

[9] Liu MH Zhou X Li GC Wang M. Exploring the regulation mechanism of TGF-β1/Smad3 pathway in COPD rats based on DCN by Miao medicine Zijiniu and Iron broom. *Chin J Ethnic Med.* (2019) 11:26–9. doi: 16041/j.cnki.cn15-1175.2019.11.018

[10] Wu J Zheng H Yao X Liu X Zhu H Yin C Comparative analysis of the compatibility effects of Danggui-Sini Decoction on a blood stasis syndrome rat model using untargeted metabolomics. *J Chromatogr B Analyt Technol Biomed Life Sci.* (2018) 1105:164–75. 10.1016/j.jchromb.2018.12.017 30594827

[11] Sun Y Chu J Geng J Guan F Zhang S Ma Y Label-free based quantitative proteomics analysis to explore the molecular mechanism of gynecological cold coagulation and blood stasis syndrome. *Anat Rec.* (2022) 306:3033–49. 10.1002/ar.25035 36136292

[12] Wei X Gao M Sheng N Yao W Bao B Cheng F Mechanism investigation of Shi-Xiao-San in treating blood stasis syndrome based on network pharmacology, molecular docking and in vitro/vivo pharmacological validation. *J Ethnopharmacol.* (2023) 301:115746. 10.1016/j.jep.2022.115746 36179951

[13] Peinado V Pizarro S Barberà J. Pulmonary vascular involvement in COPD. *Chest.* (2008) 134:808–14. 10.1378/chest.08-0820 18842913

[14] Jialin W. *Progress of Research on Drugs of the Genus Zi Jinniu. Chinese Materia Medica.* Emei: Sichuan School of Traditional Chinese Medicine (1994).

[15] Syahputra R Harahap U Dalimunthe A Nasution M Satria D. The role of flavonoids as a cardioprotective strategy against doxorubicin-induced cardiotoxicity: A Review. *Molecules.* (2022) 27:1320. 10.3390/molecules27041320 35209107 PMC8878416

[16] Kelly G. Quercetin. *Monograph Altern Med Rev.* (2011) 16:172–94. 21649459

[17] Zhou J Chuangfeng Z Lu Y. Research progress on chemical composition and pharmacological effects of truncated leaf iron broom. *Chin J Exp Formulas.* (2017) 23:228–34. 10.13422/j.cnki.syfjx.2017010228

[18] Ding J Lim I Lee H Cha W. Analysis of minerals, amino acids, and vitamin of Lespedeza cuneata. *KSBB J.* (2006) 21:414–7.

[19] Deng F Chang J Zhang J. New flavonoids and other constituents from Lespedeza cuneata. *J Asian Nat Prod Res.* (2007) 9:655–8. 10.1080/10286020600979894 17943561

[20] Jiashuo W Fangqing Z Zhuangzhuang L Weiyi J Yue S. Integration strategy of network pharmacology in Traditional Chinese Medicine: A narrative review. *J Tradit Chin Med.* (2022) 42:479–86. 10.19852/j.cnki.jtcm.20220408.003 35610020 PMC9924699

[21] Wang K Yin J Chen J Ma J Si H Xia D. Inhibition of inflammation by berberine: Molecular mechanism and network pharmacology analysis. *Phytomedicine.* (2024) 128:155258. 10.1016/j.phymed.2023.155258 38522318

[22] Liu Y Song X Dan L Tang J Jiang Y Deng C Astragali Radix: Comprehensive review of its botany, phytochemistry, pharmacology and clinical application. *Arch Pharm Res.* (2024) 47:165–218. 10.1007/s12272-024-01489-y 38493280

[23] Wenbo Z Jianwei H Hua L Lei T Guijuan C Mengfei T. The potential of flavonoids in hepatic fibrosis: A comprehensive review. *Phytomedicine.* (2024) 133:155932. 10.1016/j.phymed.2024.155932 39146877

[24] Nogales C Mamdouh Z List M Kiel C Casas A Schmidt H. Network pharmacology: Curing causal mechanisms instead of treating symptoms. *Trends Pharmacol Sci.* (2022) 43:136–50. 10.1016/j.tips.2021.11.004 34895945

[25] Qin T Wu L Hua Q Song Z Pan Y Liu T. Prediction of the mechanisms of action of Shenkang in chronic kidney disease: A network pharmacology study and experimental validation. *J Ethnopharmacol.* (2020) 246:112128. 10.1016/j.jep.2019.112128 31386888

[26] Love M Huber W Anders S. Moderated estimation of fold change and dispersion for RNA-seq data with DESeq2. *Genome Biol.* (2014) 15:550. 10.1186/s13059-014-0550-8 25516281 PMC4302049

[27] Gustavsson E Zhang D Reynolds R Garcia-Ruiz S Ryten M. ggtranscript: An R package for the visualization and interpretation of transcript isoforms using ggplot2. *Bioinformatics.* (2022) 38:3844–6. 10.1093/bioinformatics/btac409 35751589 PMC9344834

[28] Gu Z Hübschmann D. Make interactive complex heatmaps in R. *Bioinformatics.* (2022) 38:1460–2. 10.1093/bioinformatics/btab806 34864868 PMC8826183

[29] Liu P Xu H Shi Y Deng L Chen X. Potential molecular mechanisms of plantain in the treatment of gout and hyperuricemia based on network pharmacology. *Evid Based Complement Alternat Med.* (2020) 2020:3023127. 10.1155/2020/3023127 33149752 PMC7603577

[30] Chen H Boutros P. VennDiagram: A package for the generation of highly-customizable Venn and Euler diagrams in R. *BMC Bioinformatics.* (2011) 12:35. 10.1186/1471-2105-12-35 21269502 PMC3041657

[31] Yu G Wang L Han Y He Q. clusterProfiler: An R package for comparing biological themes among gene clusters. *OMICS.* (2012) 16:284–7. 10.1089/omi.2011.0118 22455463 PMC3339379

[32] Sachs M. plotROC: A Tool for Plotting ROC Curves. *J Stat Softw.* (2017) 79:2. 10.18637/jss.v079.c02 30686944 PMC6347406

[33] Robin X Turck N Hainard A Tiberti N Lisacek F Sanchez J pROC: An open-source package for R and S+ to analyze and compare ROC curves. *BMC Bioinformatics.* (2011) 12:77. 10.1186/1471-2105-12-77 21414208 PMC3068975

[34] Zhou X Minghui L Wang M Li C. Observation on the clinical efficacy of Miao medicine Zijinniu and Iron Broom on patients with phlegm and stasis mutual obstruction with qi and yin deficiency in the stable stage of COPD. *Chin Med.* (2019) 8:329–40. 10.12677/TCM.2019.85054

[35] Hu Y Cao K Wang F Wu W Mai W Qiu L Dual roles of hexokinase 2 in shaping microglial function by gating glycolytic flux and mitochondrial activity. *Nat Metab.* (2022) 4:1756–74. 10.1038/s42255-022-00707-5 36536134

[36] Krasnov G Dmitriev A Lakunina V Kirpiy A Kudryavtseva A. Targeting VDAC-bound hexokinase II: A promising approach for concomitant anti-cancer therapy. *Expert Opin Ther Targets.* (2013) 17:1221–33. 10.1517/14728222.2013.833607 23984984

[37] Fang J Luo S Lu Z. HK2: Gatekeeping microglial activity by tuning glucose metabolism and mitochondrial functions. *Mol Cell.* (2023) 83:829–31. 10.1016/j.molcel.2023.02.022 36931254

[38] He C Hao E Du C Wei W Wang X Liu T Investigating the underlying mechanisms of *Ardisia japonica* Extract’s anti-blood-stasis effect via metabolomics and network pharmacology. *Molecules.* (2023) 28:7301. 10.3390/molecules28217301 37959722 PMC10649676

[39] Leisegang MS. LET’s sponge: How the lncRNA PFL promotes cardiac fibrosis. *Theranostics.* (2018) 8:874–7. 10.7150/thno.23364 29463987 PMC5817098

[40] Cauchois R Koubi M Delarbre D Manet C Carvelli J Blasco V Early IL-1 receptor blockade in severe inflammatory respiratory failure complicating COVID-19. *Proc Natl Acad Sci U S A.* (2020) 117:18951–3. 10.1073/pnas.2009017117 32699149 PMC7430998

[41] Mujalli A Alghamdi K Nasser K Al-Rayes N Banaganapalli B Shaik N Bioinformatics insights into the genes and pathways on severe COVID-19 pathology in patients with comorbidities. *Front Physiol.* (2022) 13:1045469. 10.3389/fphys.2022.1045469 36589459 PMC9795193

[42] Lei W Jia L Wang Z Liang Z Zhao A Liu Y CC chemokines family in fibrosis and aging: From mechanisms to therapy. *Ageing Res Rev.* (2023) 87:101900. 10.1016/j.arr.2023.101900 36871782

[43] Hao W Li M Pang Y Du W Huang X. Increased chemokines levels in patients with chronic obstructive pulmonary disease: Correlation with quantitative computed tomography metrics. *Br J Radiol.* (2021) 94:20201030. 10.1259/bjr.20201030 33237823 PMC7934302

[44] Panina P Mariani M D’Ambrosio D. Chemokine receptors in chronic obstructive pulmonary disease (COPD). *Curr Drug Targets.* (2006) 7:669–74. 10.2174/138945006777435272 16787169

[45] Uwagboe I Adcock I Lo Bello F Caramori G Mumby S. New drugs under development for COPD. *Minerva Med.* (2022) 113:471–96. 10.23736/S0026-4806.22.08024-7 35142480

[46] Grimbacher B Hutloff A Schlesier M Glocker E Warnatz K Dräger R Homozygous loss of ICOS is associated with adult-onset common variable immunodeficiency. *Nat Immunol.* (2003) 4:261–8. 10.1038/ni902 12577056

[47] Di Renzo M Pasqui A Auteri A. Common variable immunodeficiency: A review. *Clin Exp Med.* (2004) 3:211–7. 10.1007/s10238-004-0027-2 15103511

[48] Wang L Zhao H Raman I Yan M Chen Q Li Q. Peripheral blood mononuclear cell gene expression in chronic obstructive pulmonary disease: miRNA and mRNA Regulation. *J Inflamm Res.* (2022) 15:2167–80. 10.2147/JIR.S337894 35392023 PMC8983057

[49] Hernandez P Duffy B Hock K Farnsworth C Schindler E Liu C. HLA-B evolutionary divergence is associated with outcomes after SARS-CoV-2 infection. *Hum Immunol.* (2022) 83:803–7. 10.1016/j.humimm.2022.09.004 36109290 PMC9464580

[50] McCluskey E Liu N Pandey A Marchetti N Sajjan U. Quercetin improves epithelial regeneration from airway basal cells of COPD patients. *Res Sq.* (2023) 25:120. 10.21203/rs.3.rs-3185241/v1 38468259 PMC10926630

[51] Boots A Haenen G Bast A. Health effects of quercetin: From antioxidant to nutraceutical. *Eur J Pharmacol.* (2008) 585:325–37. 10.1016/j.ejphar.2008.03.008 18417116

[52] Agullo G Gamet-Payrastre L Manenti S Viala C Rémésy C Chap H Relationship between flavonoid structure and inhibition of phosphatidylinositol 3-kinase: A comparison with tyrosine kinase and protein kinase C inhibition. *Biochem Pharmacol.* (1997) 53:1649–57. 10.1016/s0006-2952(97)82453-7 9264317

[53] Bals R Hiemstra P. Innate immunity in the lung: How epithelial cells fight against respiratory pathogens. *Eur Respir J.* (2004) 23:327–33. 10.1183/09031936.03.00098803 14979512

[54] Bodas M Moore A Subramaniyan B Georgescu C Wren J Freeman W Cigarette Smoke Activates NOTCH3 to promote goblet cell differentiation in human airway epithelial cells. *Am J Respir Cell Mol Biol.* (2021) 64:426–40. 10.1165/rcmb.2020-0302OC 33444514 PMC8008804

[55] Ding K Jiang W Zhan W Xiong C Chen J Wang Y The therapeutic potential of quercetin for cigarette smoking-induced chronic obstructive pulmonary disease: A narrative review. *Ther Adv Respir Dis.* (2023) 17:17534666231170800. 10.1177/17534666231170800 37154390 PMC10170608

[56] Lv Q Zhang P Quan P Cui M Liu T Yin Y Quercetin, a pneumolysin inhibitor, protects mice against Streptococcus pneumoniae infection. *Microb Pathog.* (2020) 140:103934. 10.1016/j.micpath.2019.103934 31862394

[57] Wang J Song M Pan J Shen X Liu W Zhang X Quercetin impairs Streptococcus pneumoniae biofilm formation by inhibiting sortase A activity. *J Cell Mol Med.* (2018) 22:6228–37. 10.1111/jcmm.13910 30334338 PMC6237587

[58] Rajavel T Banu Priya G Suryanarayanan V Singh S Pandima Devi K. Daucosterol disturbs redox homeostasis and elicits oxidative-stress mediated apoptosis in A549 cells via targeting thioredoxin reductase by a p53 dependent mechanism. *Eur J Pharmacol.* (2019) 855:112–23. 10.1016/j.ejphar.2019.04.051 31059712

[59] Zhang F Wang M Zha Y Zhou J Han J Zhang S. Daucosterol alleviates alcohol-induced hepatic injury and inflammation through P38/NF-κB/NLRP3 inflammasome pathway. *Nutrients.* (2023) 15:223. 10.3390/nu15010223 36615880 PMC9823995

[60] Hu L Shao C Pan L Jiang Z. Lack of STAT6 enhances murine acute lung injury through NLRP3/p38 MAPK signaling pathway in macrophages. *BMC Immunol.* (2022) 23:25. 10.1186/s12865-022-00500-9 35606692 PMC9126100

