# Supplementary material for: Ardisia japonica (Thunb.) Blume and Lespedeza cuneata G. Don may treat chronic obstructive pulmonary disease by targeting HK2 and PTAFR
Source: Front Med (Lausanne). 2025 May 6;12:1527632. doi: 10.3389/fmed.2025.1527632 (PMC12090870; doi:10.3389/fmed.2025.1527632)

Supplementary Material

# Supplementary Tables

**Supplementary Table S1:** Primer details for Reverse transcription quantitative polymerase chain reaction (RT-qPCR).

| **Primer** | **Sequence** | |
| --- | --- | --- |
| HK2 F | AACATCGTGTCACCCAGCTAA | |
| HK2 R | AGTGGGACCTCTCCGATTCA | |
| PTAFR F | CAGAGACACACGGTCACTGC | |
| PTAFR R | TCTGCACCTGGGTCTGTTTC | |
| internal reference -GAPDH F | CGAAGGTGGAGTCAACGGATTT |  |
| internal reference -GAPDH R | ATGGGTGGAATCATATTGGAAC |  |

| Ingredient id | Ingredient name | OB score | gene | Medicine |
| --- | --- | --- | --- | --- |
| HBIN012693 | 6-O-methylcatalpol | 5.802 | HK2 | ZJN |
| HBIN041495 | quercetin | 46.433 | HK2 | TSZ |
| HBIN006037 | 2-Methyl cardol | 3.562 | PTAFR | ZJN |
| HBIN016681 | Ardisinol I | 3.423 | PTAFR | ZJN |
| HBIN022771 | daucosterol | 20.631 | PTAFR | TSZ |

**Supplementary Table S2:** Effective active ingredients corresponding to key targets

ZJN: Zijinniu (*Ardisia japonica* (Thunb) Blume), TSZ:Tisaozhou (*Lespedeza cuneata* G. Don)

# Supplementary Figures

# Supplementary Fig. S1 External validation of the model based on the dataset GSE248493.

# (A): Nomogram. (B): calibration curve. (C): ROC curve.


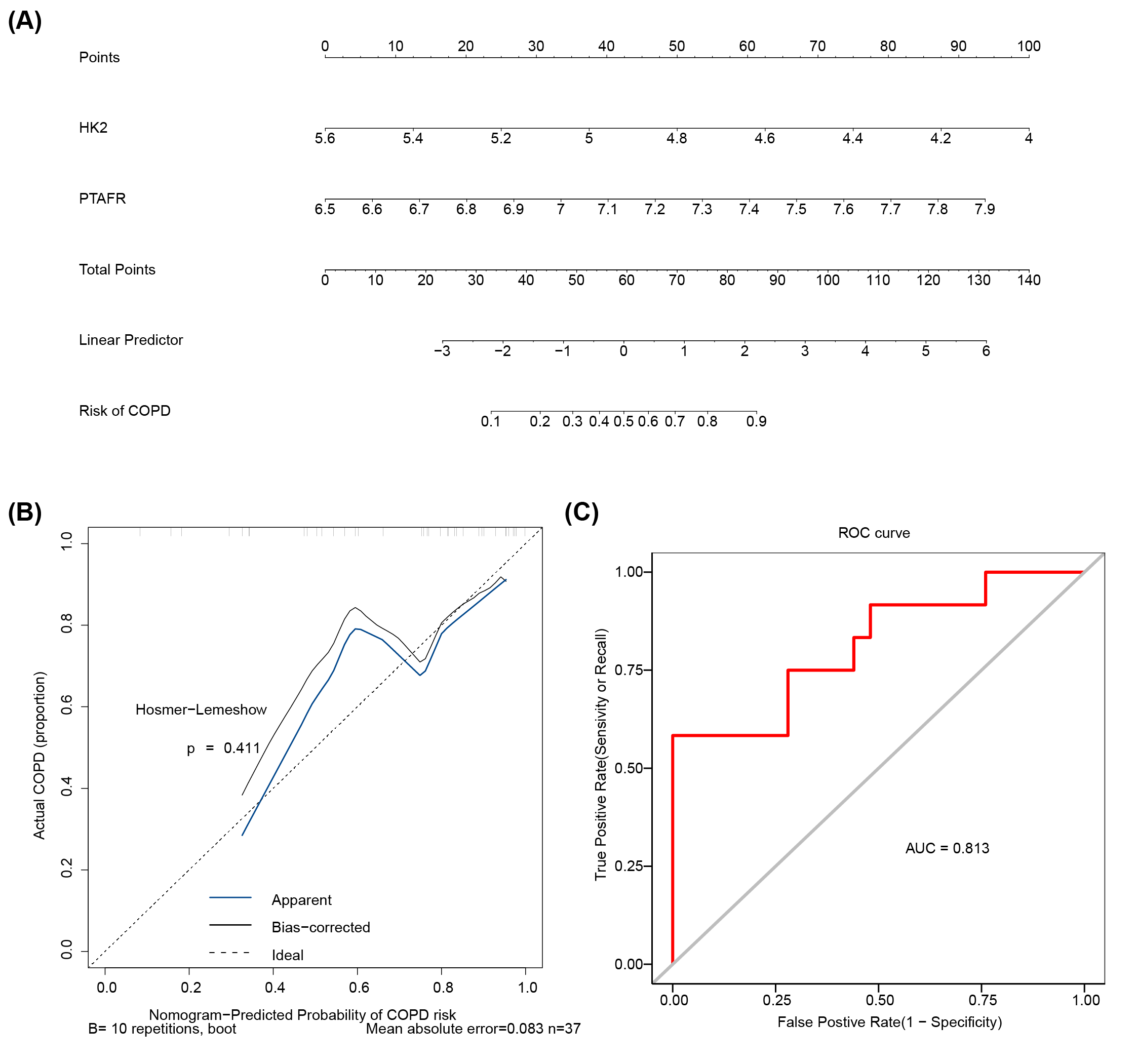

Supplement: Supplementary file 1 [file Supplementary_file_1.docx]
